# Nonparaxial Propagation of Bessel Correlated Vortex Beams in Free Space

**DOI:** 10.3390/mi14010038

**Published:** 2022-12-23

**Authors:** Nikolai I. Petrov

**Affiliations:** Scientific and Technological Centre of Unique Instrumentation of the Russian Academy of Sciences, 117342 Moscow, Russia; petrovni@mail.ru

**Keywords:** diffraction-free beams, partially coherent light, free space nonparaxial propagation, vortex beams, coherent mode representation

## Abstract

The nonparaxial propagation of partially coherent beams carrying vortices in free space is investigated using the method of decomposition of the incident field into coherent diffraction-free modes. Modified Bessel correlated vortex beams with the wavefront curvature are introduced. Analytical expressions are presented to describe the intensity distribution and the degree of coherence at different distances. The evolution of the intensity distribution during beam propagation for various source parameters is analyzed. The effects of nonparaxiality in the propagation of tightly focused coherent vortex beams are analyzed.

## 1. Introduction

Coherent properties of fields must be taken into account in many problems of wave propagation in free space and in inhomogeneous media. This is due to the fact that real sources (laser, LEDs, etc.) generate partially coherent radiation, and purely coherent sources, as a rule, are not implemented in practice. Partially coherent beams are useful in remote sensing, ghost imaging, optical communication, particle trapping, etc.

The theory of propagation of partially coherent waves in free space, as well as in inhomogeneous media, has now been developed quite fully [1,2,3,4]. The conventional Gaussian-Schell-model partially coherent beam was studied mainly until the recent past. Partially coherent sources with specific propagation properties that lead to the formation of highly directional light beams were considered in [5,6,7].

In recent decades, partially coherent vortex beams have aroused great interest due to their unique propagation properties and hidden correlation features [8,9,10,11,12,13,14,15,16,17,18]. It was shown in [9] that the shape of a focused beam can be controlled by changing the initial spatial coherence length. Recently, a variety of partially coherent beams with extraordinary properties has been presented [15,16,17,18]. In [8], a sufficient condition was proposed for devising a genuine correlation function of a partially coherent beam. Based on this, various partially coherent vortex beams with nonconventional correlation functions were introduced. A new class of Laguerre–Christoffel–Darboux sources emitting shape-invariant beams is introduced in [19,20]. Vortex partially coherent beams carrying orbital angular momentum (OAM) are useful in optical manipulation and trapping, optical machining, optical communication, quantum information, and optical microscopy.

Currently, various beams are being studied, ranging from scalar vortex beams to vector vortex beams [16,17,18,21,22,23]. It was found that the polarization properties of vector partially coherent beams change during propagation in free space.

In [24], a new family of partially coherent beams was introduced, the cross-spectral density function (CSD) of which is represented as an incoherent superposition of fully coherent Laguerre–Gauss modes of arbitrary order. Recently, partially coherent vortex beams have been introduced, which are an incoherent superposition of diffraction-free Bessel modes with the same helical wavefront [25,26]. In [27], an experimental implementation of the Bessel-correlated beams as a superposition of coherent modes generated using a spatial light modulator was presented. Modeling of beam propagation with Bessel correlation through time-correlated atmospheric turbulence was presented in [28].

The propagation characteristics of a partially coherent beam in free space and an inhomogeneous medium have been studied in recent decades [29,30,31,32,33,34,35]. However, most publications are devoted to the analysis of beam characteristics in the paraxial approximation. Nonparaxial propagation properties of partially coherent Lorentz–Gauss and four-petal Gaussian vortex beams on the basis of the Rayleigh–Sommerfeld diffraction integral were investigated in [36,37]. In [38,39] nonparaxial propagation of vector partially coherent beams are considered. It is well known that nonparaxial effects significantly affect the characteristics of tightly focused beams [40,41,42,43,44,45,46,47,48,49,50,51]. Tightly focused beams find important applications in optical data storage, microscopy, particle trapping, etc. Integral methods are widely used to analyze the evolution of structured beams in free space. Calculations are usually performed numerically on a submicron scale due to the existence of high-frequency oscillatory terms in the Rayleigh–Sommerfeld integral. Therefore, other time-saving modeling approaches are needed to facilitate the solution of the problem.

In this paper, the nonparaxial propagation of partially coherent vortex light beams with Bessel correlation in free space is investigated using the mode decomposition method. This approach significantly reduces the simulation time, since there is no need to calculate complex Rayleigh–Sommerfeld diffraction integrals. A modified cross-spectral density function describing the Bessel-correlated beams with an additional parameter is introduced. The mode expansion coefficients are obtained analytically. Analytical expressions for the intensity distribution and degree of coherence are presented. The influence of nonparaxiality effects on the field intensity distributions in the axial and radial directions in the focusing plane is analyzed.

## 2. Cross-Spectral Density Function

The coherence properties of the field radiated by the source are described by the cross-spectral density (CSD) function $W\left(r_{1},\;r_{2},0\right)$ [2] which is defined as the second-order correlations of the field at two different points.

The degree of coherence is defined by the CSD function [2]:

(1) $$\gamma \left(r_{1},r_{2},0\right)=\frac{W\left(r_{1},r_{2},0\right)}{\sqrt{I\left(r_{1},0\right)I\left(r_{2},0\right)}}$$

where $I\left(r,0\right)=W\left(r,r,0\right)$ is the average intensity at coincident points.

Partially coherent vortex beams with various CSD were considered in the past decades. The CSD function of the Gaussian-Schell-model vortex beam in the source plane is given by [16,17,18]:

(2) $$W\left(r_{1},r_{2},\varphi_{1},\;\varphi_{2}\right)=exp\left\{-\frac{r_{1}^{2}+r_{2}^{2}}{4w_{0}^{2}}-\frac{r_{1}^{2}+r_{2}^{2}-2r_{1}r_{2}\cos\left(\varphi_{1}-\varphi_{2}\right)}{2r_{0}^{2}}+il\left(\varphi_{1}-\varphi_{2}\right)\right\}$$

where *r* and *φ* are the radial and polar angle coordinates, respectively, $w_{0}$ is the beam width, $r_{0}$ is the coherence length.

The Laguerre–Gaussian correlated Schell-model vortex beam was experimentally demonstrated in [52]:

(3) $$W\left(r_{1},r_{2},\varphi \right)=exp\left[-\frac{r_{1}^{2}+r_{2}^{2}}{4w_{0}^{2}}-\frac{{\left(r_{1}-r_{2}\right)}^{2}}{2r_{0}^{2}}\right]L_{p}^{0}\left[\frac{{\left(r_{1}-r_{2}\right)}^{2}}{2r_{0}^{2}}\right]exp\left[il\left(\varphi_{1}-\varphi_{2}\right)\right]$$

where $L_{p}^{0}\left[z\right]$ denotes the Laguerre polynomial.

In [25], the cross-spectral density of a partially coherent diffraction-free vortex beam with a Bessel-mode structure is considered:

(4) $$W\left(r_{1},r_{2}\right)=e^{ik\left(\varphi_{2}-\varphi_{1}\right)}\underset{n}{\sum }J_{l}\left(\mu_{nl}\frac{r_{1}}{R_{0}}\right)J_{l}\left(\mu_{nl}\frac{r_{2}}{R_{0}}\right)$$

Below we consider the vortex Bessel–Gauss correlated beams [24]:

(5) $$W\left(r_{1},r_{2}\right)=\frac{A\xi^{-l/2}}{\left(1-\xi \right)}exp\left[-\frac{1+\xi }{1-\xi }\frac{r_{1}^{2}+r_{2}^{2}}{w^{2}}\right]I_{l}\left(\frac{4\sqrt{\xi }}{1-\xi }\frac{r_{1}r_{2}}{w^{2}}\right)exp\left[-il\left(\varphi_{1}-\varphi_{2}\right)\right]$$

where $\xi =\frac{r_{0}^{4}}{w^{4}}{\left[{\left(1+\frac{w^{4}}{r_{0}^{4}}\right)}^{1/2}-1\right]}^{2}$, w is the spot size at the waist of the beam, $r_{0}$ is the coherence length.

Note that when $r_{0}\to \infty$ (fully coherent case) ξ→0 and when $r_{0}\to 0$ (completely incoherent case) ξ→1.

The intensity can be calculated from the CSD function at coincident points $r_{1}=r_{2}$, i.e.,

(6) $$I\left(r\right)=W\left(r,r\right)$$

**Figure 1:**
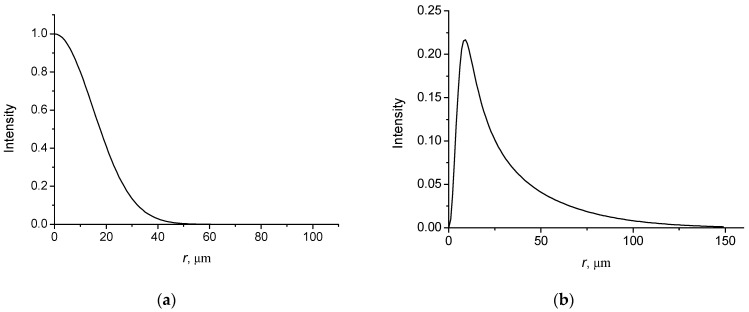
Intensity distributions of BG vortex beam with *l* = 0. *w* = 30 µm; (**a**) ξ = 0.02; *r*_coh_ = 100 µm; (**b**) ξ = 0.80; *r*_coh_ = 10 µm.

In Figure 1, the intensity distributions of the field with *l* = 0 as function of the radial distance are shown for the spatially high coherent (ξ = 0.02) and nearly incoherent (*ξ* = 0.80) cases.

**Figure 2:**
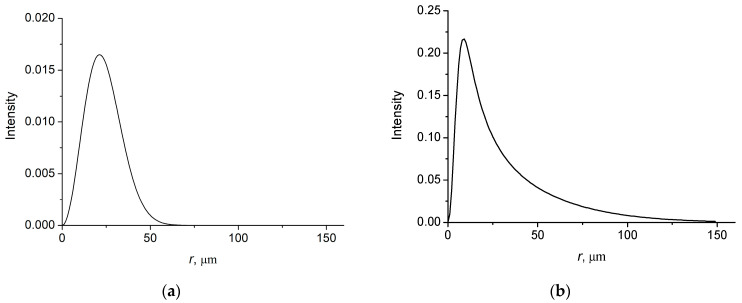
Intensity distributions of BG vortex beam with *l* = 1. *w* = 30 µm; (**a**) ξ = 0.02; *r*_coh_ = 100 µm; (**b**) ξ = 0.80; *r*_coh_ = 10 µm.

In Figure 2, the intensity distributions of the field with *l* = 1 as function of the radial distance are shown for the spatially coherent and incoherent cases.

It is seen from the figures that the intensity distributions of the coherent beam are symmetric with the width of the order of *w*. For an incoherent beam, the intensity distribution is asymmetric with a long tail, the length of which is of the order of coherence length *r*_coh_. A characteristic diffraction angle of a coherent beam in paraxial regime is defined by $\theta_{d}\;\sim \;\lambda /w$, while for an incoherent beam it is defined by the coherence length, i.e., $\theta_{d}\;\sim \;\lambda /r_{coh}.$ Note that for a nearly incoherent beam $r_{coh}\ll w$.

## 3. Coherent Mode Representation

Using the coherent mode decomposition, the cross-spectral density function of a source can be represented as [2]:

(7) $$W\left(r_{1},\;r_{2}\right)=\underset{n}{\sum }\lambda_{n}\Psi_{n}^{*}\left(r_{1}\right)\Psi_{n}\left(r_{2}\right)$$

where $\lambda_{n}$ and $\Psi_{n}\left(r\right)$ are the eigenvalues and eigenfunctions of the Fredholm integral equation of the second kind

(8) $$\int W\left(r_{1},\;r_{2}\right)\Psi_{n}\left(r_{1}\right)d^{2}r_{1}=\lambda_{n}\Psi_{n}\left(r_{2}\right)$$

Note that all the eigenvalues $\lambda_{n}$ are non-negative, and the eigenfunctions $\Psi_{n}\left(r\right)$ form an orthogonal set.

If $\Psi_{n}\left(r\right)$ are chosen to be the coherent Laguerre–Gauss modes

(9) $$\Psi_{nl}\left(r,\varphi \right)=\frac{1}{w_{0}}{\left(\frac{2n!}{\pi \left(n+l\right)!}\right)}^{1/2}{\left(\frac{\sqrt{2}r}{w_{0}}\right)}^{l}e^{-r^{2}/w_{0}^{2}}L_{n}^{l}\left(\frac{2r^{2}}{w_{0}^{2}}\right)e^{il\varphi }$$

then the CSD takes a closed form (5) [24].

The eigenvalues in Equation (7) are equal to

(10) $$\lambda_{nl}=\frac{n!}{\left(n+l\right)!}\xi^{n}$$

### 3.1. Nonparaxial Propagation in Free Space

Below, we consider the nonparaxial evolution of vortex Bessel–Gauss correlated CSD.

To do this, instead of Laguerre–Gauss modes [24] in Equation (5), we represent the CSD function as an incoherent superposition of coherent orthogonal diffraction-free vortex Bessel modes which are the solutions of Helmholtz equation.

The evolution of the LG modes is determined by the expression

(11) $$\Psi_{nl}\left(r,\varphi ,z\right)=\underset{pl}{\sum }a_{pn}\psi_{pl}\left(r,\varphi \right)e^{i\beta_{pl}z}$$

where $\psi_{pl}\left(r,\varphi \right)$ are the modal solutions and $\beta_{pl}$ are the propagation constants of the Bessel modes defined from the Helmholtz equation.

The normalized Bessel functions with radial *p* and azimuthal *l* indices can be considered as the modal solutions of Helmholtz equation within the effective depth of field [53]:

(12) $$\psi_{pl}\left(r,\varphi \right)=\frac{J_{l}\left(\mu_{pl}\frac{r}{R_{0}}\right)\exp(il\varphi )}{\left(\sqrt{\pi }R_{0}J_{l+1}\left(\mu_{pl}\right)\right)}$$

where $\mu_{1},\mu_{2},\ldots$ are the positive zeros of the Bessel function $J_{l}\left(z\right)$.

It follows from the orthogonality condition for Bessel functions [54].

(13) $$\int_{0}^{R_{0}}J_{m}\left(\mu_{i}r/R_{0}\right)J_{m}\left(\mu_{j}r/R_{0}\right)\rho d\rho =\frac{R_{0}^{2}}{2}{\left[J_{m+1}\left(\mu_{i}\right)\right]}^{2}\delta_{ij}$$

that these modes satisfy the equation

(14) $$\int_{0}^{2\pi }\int_{0}^{R_{0}}\psi_{pl}^{*}\left(\rho ,\varphi \right)\psi_{pl}\left(\rho ,\varphi \right)\rho d\rho d\varphi =1$$

The solutions (12) form a complete set of mutually orthogonal functions in the given interval [0, *R*_0_]. Hence, any field in the initial plane *z* = 0 can be decomposed into these modal solutions.

Solutions (12) are the non-diffractive Bessel beams with radial and azimuthal indices which are propagation-invariant in free space. Note that the non-diffractive scalar Bessel beams in free space were considered in [55], where a narrow annular slit in the screen together with a spherical lens located at a focal distance from it is proposed to create a zero-order Bessel beam. In [56], the decomposition of the field into Bessel modes was used when considering the scattering of light by small particles. In [57], the mode properties of Bessel beams of nonzero order in free space are discussed. Phase optical components are proposed in [58] for the generation of free-space Bessel modes. In [59,60], the amplitude components of the vector non-diffractive beams were obtained as solutions to the vector Helmholtz wave equation. It was shown in [53] that the normalized Bessel beams (12) with radial indices are the solutions of the scalar Helmholtz equation.

The modal coefficients $a_{pn}$ are determined from the integration

(15) $$a_{pn}=\iint \Psi_{nl}^{*}\left(r,\varphi ,0\right)\psi_{pl}\left(r,\varphi \right)rdrd\varphi$$

Using the integral [61]

(16) $$\overset{\infty }{\underset{0}{\int }}x^{l/2}e^{-px}J_{\nu }\left(b\sqrt{x}\right)L_{n}^{l}\left(cx\right)dx={\left(\frac{b}{2}\right)}^{l}\frac{{\left(p-c\right)}^{n}}{p^{n+l+1}}exp\left(-\frac{b^{2}}{4p}\right)L_{n}^{l}\left(\frac{b^{2}c}{4pc-4p^{2}}\right)$$

we find that

(17) $$a_{pn}=B_{0}{\left(-1\right)}^{n}{\left(\frac{\mu_{pl}}{2R_{0}}\right)}^{l}exp\left(-\frac{\mu_{pl}^{2}w_{0}^{2}}{4R_{0}^{2}}\right)L_{n}^{l}\left(\frac{\mu_{pl}^{2}w_{0}^{2}}{2R_{0}^{2}}\right),$$

where $B_{0}={\left(2w_{0}^{2}\right)}^{\left(l+1\right)/2}\sqrt{\frac{n!}{\left(n+1\right)!}}\frac{1}{R_{0}J_{l+1}\left(\mu_{pl}\right)}$.

The evolution of the intensity distribution is given by

(18) $$I\left(r,z\right)=W\left(r,r,z\right)=\underset{nl}{\sum }\lambda_{nl}\Psi_{nl}^{*}\left(r,z\right)\Psi_{nl}\left(r,z\right)=\underset{n}{\sum }\lambda_{n}\underset{pp^{\prime }}{\sum }a_{p}^{*}a_{p^{\prime }}\psi_{p}^{*}\psi_{p^{\prime }}cos\left[\left(\beta_{p}-\beta_{p^{\prime }}\right)z\right]$$

where

$$\lambda_{n}=\frac{n!}{\left(n+l\right)!}\xi^{n};\;\xi =\frac{r_{0}^{4}}{w_{0}^{4}}{\left[{\left(1+\frac{w_{0}^{4}}{r_{0}^{4}}\right)}^{1/2}-1\right]}^{2};$$

$$\Psi_{nl}\left(r,\varphi \right)=\frac{1}{w_{0}}{\left(\frac{2n!}{\pi \left(n+l\right)!}\right)}^{1/2}{\left(\frac{\sqrt{2}r}{w_{0}}\right)}^{l}e^{-r^{2}/w_{0}^{2}}L_{n}^{l}\left(\frac{2r^{2}}{w_{0}^{2}}\right)e^{il\varphi },$$

$$\psi_{pl}\left(r,\varphi \right)=\frac{J_{l}\left(\mu_{pl}\frac{r}{R_{0}}\right)\exp(il\varphi )}{\left(\sqrt{\pi }R_{0}J_{l+1}\left(\mu_{pl}\right)\right)};$$

$$a_{p}=B_{0}{\left(-1\right)}^{n}{\left(\frac{\mu_{pl}}{2R_{0}}\right)}^{l}\frac{q^{*n}}{q^{n+l+1}}exp\left(-\frac{\mu_{pl}^{2}}{4R_{0}^{2}q}\right)L_{n}^{l}\left(\frac{\mu_{pl}^{2}}{2w_{0}^{2}R_{0}^{2}{\left|q\right|}^{2}}\right);\;B_{0}={\left(\frac{2}{w_{0}^{2}}\right)}^{\left(l+1\right)/2}\sqrt{\frac{n!}{\left(n+1\right)!}}\frac{1}{R_{0}J_{l+1}\left(\mu_{pl}\right)};$$

The evolution of the CSD has the form

(19) $$W\left(r_{1},r_{2},z\right)=\underset{nl}{\sum }\lambda_{nl}\underset{pp^{\prime }}{\sum }c_{pl}c_{p^{\prime }l}J_{l}\left(\mu_{p}r_{1}/R_{0}\right)J_{l}\left(\mu_{p^{\prime }}r_{2}/R_{0}\right)e^{i\left(\beta_{pl}-\beta_{p^{\prime }l}\right)z}$$

where $c_{pl}=\frac{a_{p}}{\sqrt{\pi }R_{0}J_{l+1}\left(\mu_{pl}\right)}$.

For the particular case of the CSD with *l* = 0 we have

(20) $$I\left(r,0\right)=W\left(r,r,0\right)=A_{0}exp\left\{-\frac{1+\xi }{1-\xi }\frac{2r^{2}}{r_{0}^{2}}\right\}I_{0}\left(\frac{4\sqrt{\xi }}{\left(1-\xi \right)}\frac{r^{2}}{r_{0}^{2}}\right)=\underset{n}{\sum }\lambda_{n0}\Psi_{n0}^{*}\Psi_{n0}=\underset{n}{\sum }\lambda_{n}\underset{pp^{\prime }}{\sum }a_{pn}^{*}a_{p^{\prime }n}\psi_{p}^{*}\psi_{p^{\prime }}$$

where $\lambda_{n}=\xi^{n}$; $\xi =\frac{r_{0}^{4}}{w_{0}^{4}}{\left[{\left(1+\frac{w_{0}^{4}}{r_{0}^{4}}\right)}^{1/2}-1\right]}^{2};$ $a_{p}=B_{0}{\left(-1\right)}^{n}w_{0}^{2}exp\left(-\frac{\mu_{p0}^{2}w_{0}^{2}}{4R_{0}^{2}}\right)L_{n}^{0}\left(\frac{\mu_{p0}^{2}w_{0}^{2}}{2R_{0}^{2}}\right)$; $B_{0}=\frac{\sqrt{2}}{w_{0}}\frac{1}{R_{0}J_{1}\left(\mu_{p0}\right)}$.

Note that the values of $\lambda_{n}$ decrease with the mode number *n*, and the number of modes with a noticeable contribution increases significantly with decreasing coherence radius $r_{0}$.

The intensity profile change at propagation is defined by

(21) $$I\left(r,z\right)=\underset{n}{\sum }\lambda_{n}\underset{pp^{\prime }}{\sum }a_{p}^{*}a_{p^{\prime }}\psi_{p}^{*}\psi_{p^{\prime }}cos\left[\left(\beta_{p}-\beta_{p^{\prime }}\right)z\right],$$

where $\beta_{p0}=k_{0}{\left[1-{\left(\frac{\mu_{p0}}{k_{0}R_{0}}\right)}^{2}\right]}^{1/2}$.

Analogously, for the intensity of the source with *l* = 1 we have

(22) $$I\left(r,0\right)=W\left(r,r,0\right)=A_{0}exp\left\{-\frac{1+\xi }{1-\xi }\frac{2r^{2}}{r_{0}^{2}}\right\}I_{1}\left(\frac{4\sqrt{\xi }}{\left(1-\xi \right)}\frac{r^{2}}{r_{0}^{2}}\right)=\underset{n}{\sum }\lambda_{n1}\Psi_{n1}^{*}\Psi_{n1}=\underset{n}{\sum }\lambda_{n}\underset{pp^{\prime }}{\sum }a_{pn}^{*}a_{p^{\prime }n}\psi_{p}^{*}\psi_{p^{\prime }}$$

where $\lambda_{n}=\frac{n!}{\left(n+1\right)!}\xi^{n}$; $\xi =\frac{r_{0}^{4}}{w_{0}^{4}}{\left[{\left(1+\frac{w_{0}^{4}}{r_{0}^{4}}\right)}^{1/2}-1\right]}^{2};$

$$a_{p}=B_{0}{\left(-1\right)}^{n}\frac{\mu_{p1}w_{0}^{4}}{2R_{0}}exp\left(-\frac{\mu_{p1}^{2}w_{0}^{2}}{4R_{0}^{2}}\right)L_{n}^{1}\left(\frac{\mu_{p1}^{2}w_{0}^{2}}{2R_{0}^{2}}\right);\;B_{0}=\frac{2}{w_{0}^{2}}\sqrt{\frac{n!}{\left(n+1\right)!}}\frac{1}{R_{0}J_{2}\left(\mu_{p1}\right)}.$$

The intensity change with distance is determined by the expression

(23) $$I\left(r,z\right)=\underset{n}{\sum }\lambda_{n}\underset{pp^{\prime }}{\sum }c_{p}^{*}c_{p^{\prime }}\psi_{p}^{*}\psi_{p^{\prime }}cos\left[\left(\beta_{p}-\beta_{p^{\prime }}\right)z\right]$$

where $\beta_{p1}=k_{0}{\left[1-{\left(\frac{\mu_{p1}}{k_{0}R_{0}}\right)}^{2}\right]}^{1/2}$.

### 3.2. Modified Partially Coherent Vortex Bessel–Gauss Beams

Consider a partially coherent Bessel–Gauss beam with the wavefront curvature radius *R*_f_ in the plane *z* = 0.

(24) $$W\left(r_{1},r_{2}\right)=\frac{A\xi^{-l/2}}{\left(1-\xi \right)}exp\left[-\frac{1+\xi }{1-\xi }\frac{r_{1}^{2}+r_{2}^{2}}{2w^{2}}-\frac{ik_{0}}{2R_{f}}\left(r_{2}^{2}-r_{1}^{2}\right)\right]I_{l}\left(\frac{4\sqrt{\xi }}{1-\xi }\frac{r_{1}r_{2}}{w^{2}}\right)exp\left[-il\left(\varphi_{1}-\varphi_{2}\right)\right]$$

where $R_{f}$ is the wavefront curvature, $k_{0}=2\pi /\lambda$ is the wavenumber.

Unlike CSD (5), here, we introduced the new parameter—the wavefront curvature radius *R*_f_. In contrast to the conventional LG modes, here, we consider generalized Laguerre–Gauss modes with spherical wavefrontss the eigenfunctions

(25) $$\Psi_{nl}\left(r,\varphi \right)=\frac{1}{w_{0}}{\left(\frac{2n!}{\pi \left(n+l\right)!}\right)}^{1/2}{\left(\frac{\sqrt{2}r}{w_{0}}\right)}^{l}e^{-r^{2}/w_{0}^{2}-ikr^{2}/2R_{f}}L_{n}^{l}\left(\frac{2r^{2}}{w_{0}^{2}}\right)e^{il\varphi }$$

The intensity distribution in this case is expressed by

(26) $$I\left(r,z\right)=\underset{n}{\sum }\lambda_{n}\underset{pp^{\prime }}{\sum }a_{p}^{*}a_{p^{\prime }}\psi_{p}^{*}\psi_{p^{\prime }}cos\left[\left(\beta_{p}-\beta_{p^{\prime }}\right)z\right]$$

where $\lambda_{n}=\frac{n!}{\left(n+l\right)!}\xi^{n}$; $\xi =\frac{r_{0}^{4}}{w_{0}^{4}}{\left[{\left(1+\frac{w_{0}^{4}}{r_{0}^{4}}\right)}^{1/2}-1\right]}^{2};$ $\psi_{pl}\left(r,\varphi \right)=\frac{J_{l}\left(\mu_{pl}\frac{r}{R_{0}}\right)\exp(il\varphi )}{\left(\sqrt{\pi }R_{0}J_{l+1}\left(\mu_{pl}\right)\right)}$;

$$a_{p}=B_{0}{\left(-1\right)}^{n}{\left(\frac{\mu_{pl}}{2R_{0}}\right)}^{l}\frac{q^{*n}}{q^{n+l+1}}exp\left(-\frac{\mu_{pl}^{2}w_{0}^{2}}{4R_{0}^{2}q}\right)L_{n}^{l}\left(\frac{\mu_{pl}^{2}}{2w_{0}^{2}R_{0}^{2}{\left|q\right|}^{2}}\right);\;B_{0}={\left(\frac{2}{w_{0}^{2}}\right)}^{\left(l+1\right)/2}\sqrt{\frac{n!}{\left(n+1\right)!}}\frac{1}{R_{0}J_{l+1}\left(\mu_{pl}\right)};$$

$$q=\frac{1}{w^{2}}+\frac{ik_{0}}{2R_{f}};\;q^{*}=\frac{1}{w^{2}}-\frac{ik_{0}}{2R_{f}}.$$

The evolution of the modified CSD is given by

(27) $$W\left(r_{1},r_{2},z\right)=\underset{nl}{\sum }\lambda_{nl}\underset{pp^{\prime }}{\sum }c_{pl}c_{p^{\prime }l}J_{l}\left(\mu_{p}r_{1}/R_{0}\right)J_{l}\left(\mu_{p^{\prime }}r_{2}/R_{0}\right)e^{i\left(\beta_{pl}-\beta_{p^{\prime }l}\right)z}$$

where $c_{pl}=\frac{a_{p}}{\sqrt{\pi }R_{0}J_{l+1}\left(\mu_{pl}\right)}$.

## 4. Propagation of Coherent Vortex Bessel–Gauss Beams

Below, we apply the mode decomposition method to analyze the propagation of coherent vortex BG beams in free space.

Consider the incident beam at *z* = 0 with Gaussian and Bessel–Gauss (BG) spatial distributions of the intensity:

(28) $$\vec{E}\left(r,\varphi ,0\right)=\sqrt{\frac{2}{\pi }}\frac{1}{w_{0}}\exp\left(-r^{2}/w_{0}^{2}\right),$$

(29) $$\vec{E}\left(r,\varphi ,0\right)=A_{0}\exp\left(-r^{2}/w_{0}^{2}\right)J_{0}\left(\gamma r\right)$$

(30) $$\vec{E}\left(r,\varphi ,0\right)=A_{0}\exp\left(-r^{2}/w_{0}^{2}\right)J_{0}\left(\gamma r\right)$$

where $w_{0}$ is the radius of a Gaussian beam, $A_{0}=\sqrt{\frac{2}{\pi }}\frac{1}{w_{0}}\exp\left(\frac{1}{8}\gamma^{2}w_{0}^{2}\right){\left[I_{0}\left(\frac{1}{4}\gamma^{2}w_{0}^{2}\right)\right]}^{-1/2},$ $B_{0}=\sqrt{\frac{2}{\pi }}\frac{1}{w_{0}}\exp\left(\frac{1}{8}\gamma^{2}w_{0}^{2}\right){\left[I_{l}\left(\frac{1}{4}\gamma^{2}w_{0}^{2}\right)\right]}^{-1/2}$, $w_{B}/\mu_{1}=\gamma^{-1}$ is the effective width of the BG beam.

The modal amplitude coefficients for these incident beams can be calculated analytically. The expressions for modal coefficients have the form:

(31) $$a_{pl}=\frac{\sqrt{2}w_{0}}{R_{0}J_{1}\left(\mu_{p}\right)}\exp\left\{-\frac{w_{0}^{2}\mu_{p}^{2}}{4R_{0}^{2}}\right\}$$

(32) $$a_{pl}=\frac{A_{0}\sqrt{\pi }w_{0}^{2}}{R_{0}J_{1}\left(\mu_{p}\right)}\exp\left[-\left(\alpha^{2}+\gamma^{2}\right)w_{0}^{2}/4\right]I_{0}\left(\frac{\alpha \gamma }{2}w_{0}^{2}\right)$$

(33) $$a_{pl}=\frac{B_{0}\sqrt{\pi }w_{0}^{2}}{R_{0}J_{l+1}\left(\mu_{p}\right)}\exp\left[-\left(\alpha^{2}+\gamma^{2}\right)w_{0}^{2}/4\right]I_{l}\left(\frac{\alpha \gamma }{2}w_{0}^{2}\right)$$

where $\mu_{p}$ are the positive zeros of the Bessel functions, and $I_{0}\left(z\right)$ and $I_{1}\left(z\right)$ are the modified Bessel functions of the first kind.

The evolution of the incident field is determined by the expression

(34) $$\vec{E}\left(r,\varphi ,z\right)=\underset{pl}{\sum }a_{pl}\Psi_{pl}\left(r,\varphi ,0\right)e^{i\beta_{pl}z}$$

where $\beta_{pl}=k_{0}{\left[1-{\left(\frac{\mu_{pl}}{k_{0}R_{0}}\right)}^{2}\right]}^{1/2}$ are the propagation constants of the modes (12).

### 4.1. Bessel–Gauss Beam with l = 0

Consider a Bessel–Gauss beam with *l* = 0 at the source plane *z* = 0:

(35) $$E\left(r,0\right)=A_{0}exp\left(-\frac{r^{2}}{w_{0}^{2}}\right)J_{0}\left(\gamma r\right)$$

where $A_{0}=\sqrt{\frac{2}{\pi }}\frac{1}{w_{0}}\exp\left(\frac{1}{8}\gamma^{2}w_{0}^{2}\right){\left[I_{0}\left(\frac{1}{4}\gamma^{2}w_{0}^{2}\right)\right]}^{-1/2}$, $w_{B}/\mu_{1}=\gamma^{-1}$ is the effective width of the BG beam.

**Figure 3:**
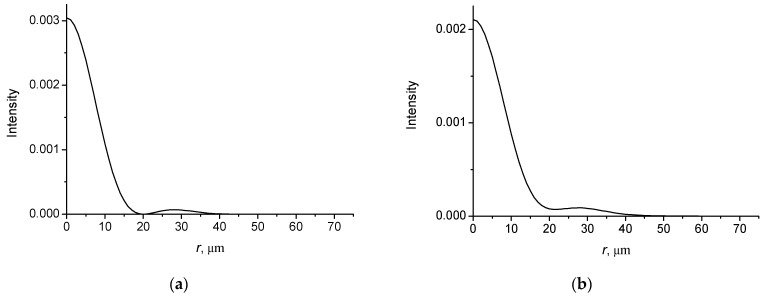

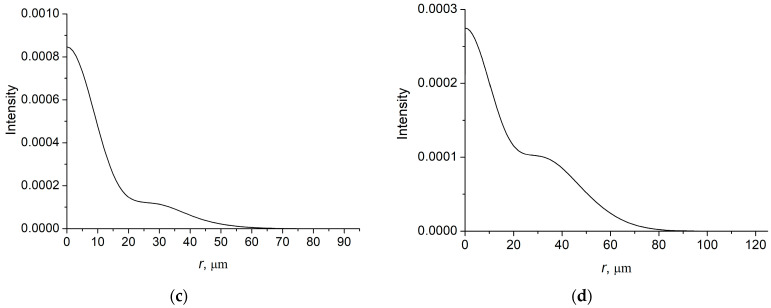
Intensity distributions of BG beam with *l* = 0; $w_{0}$ = 30 mm; *w_B_* = 20 mm; *l* = 0.63 µm. (**a**) *z* = 0; (**b**) *z* = 1000 µm; (**c**) *z* = 2000 µm; (**d**) *z* = 3000 µm.

In Figure 3, the intensity distributions of Bessel–Gauss beam with *l* = 0 as function of the radial distance are presented at different propagation distances.

It follows that the diffraction spreading of the BG beam is significantly less than for Gaussian beam, i.e., the BG beams have a sharp radiation pattern. Note the intensity distributions are in good agreement with the results of numerical simulations obtained using the Fresnel diffraction integral [62].

### 4.2. Bessel–Gauss Beam with l = 1

Let us now consider a Bessel–Gauss beam with *l* = 1 in the plane of the source *z* = 0:

(36) $$E\left(r,0\right)=B_{0}exp\left(-\frac{r^{2}}{w_{0}^{2}}\right)J_{1}\left(\gamma r\right)\;$$

where $B_{0}=\sqrt{\frac{2}{\pi }}\frac{1}{w_{0}}\exp\left(\frac{1}{8}\gamma^{2}w_{0}^{2}\right){\left[I_{1}\left(\frac{1}{4}\gamma^{2}w_{0}^{2}\right)\right]}^{-1/2}$*,* $w_{B}/\mu_{1}=\gamma^{-1}$ is the effective width of the BG beam.

The total power P of the incident beam is normalized, i.e.,

$$P=\iint {\left|E\left(r,0\right)\right|}^{2}rdrd\varphi =1$$

**Figure 4:**
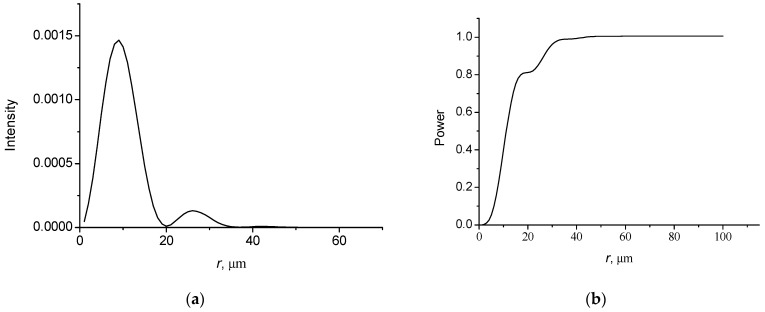

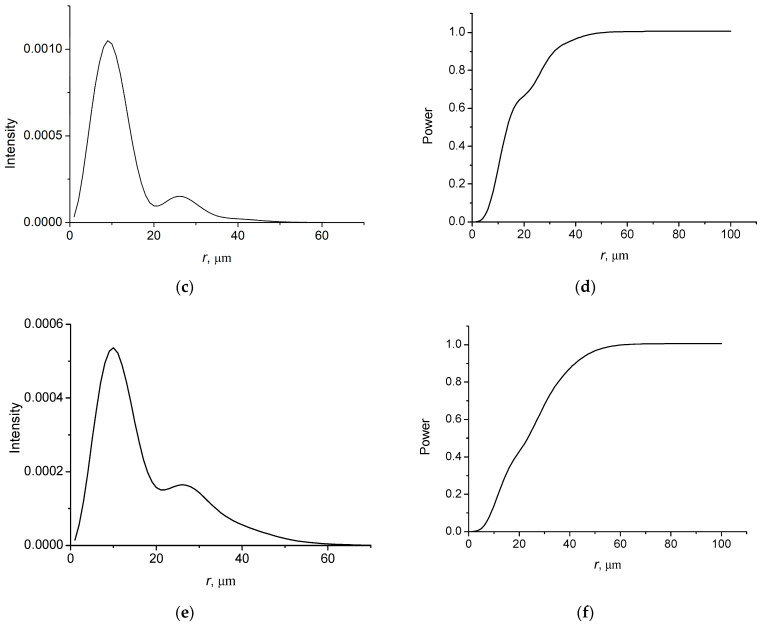
Intensity distributions of BG vortex beam with *l* = 1. $w_{0}$ = 30 µm, *w_B_* = 20 µm, *λ* = 0.63 µm. (**a**,**b**) *z* = 0; (**c**,**d**) *z* = 500 µm; (**e**,**f**) *z* = 1000 µm.

In Figure 4, the intensity distributions (a, c, e) and powers (b, d, f) of Bessel–Gauss beam with *l* = 1 as function of the radial distance are presented at different propagation distances.

It follows from simulations that the beam profile retains its original shape when propagating over long distances.

### 4.3. Nonparaxial Propagation and Focusing of a Gaussian Beam

The propagation of a Gaussian beam in free space has long been well studied using various methods, including analytical, asymptotic and numerical approaches to integration. Usually, the problem is solved by evaluation of diffraction integrals. However, numerical simulation of diffraction integrals is often time consuming, and asymptotic methods have been developed for calculations. A hybrid integration method combining numerical integration with asymptotic methods was proposed in [63]. In [64], the accuracy and computational savings of this hybrid technology were examined.

Below, we show that the effects resulting from the diffraction-free mode decomposition method are in good agreement with the known results.

Consider the incident field at *z* = 0:

(37) $$E\left(r,0\right)=A_{0}exp\left(-\frac{r^{2}}{w^{2}}-\frac{ik_{0}}{2R_{f}}r^{2}\right),$$

where $A_{0}=\sqrt{\frac{2}{\pi }}\frac{1}{w}$; $k_{0}=\frac{2\pi }{\lambda }$; $R_{f}$ is the wavefront curvature.

Evolution of the field with distance is given by

(38) $$E\left(r,z\right)=\underset{p}{\sum }a_{p}\psi_{p}\left(r\right)e^{i\beta_{p}z},$$

where $a_{p}=\frac{\sqrt{2}}{wR_{0}J_{1}\left(\mu_{p}\right)}\frac{1}{q}\exp\left\{-\frac{{\left(\mu_{p}/R_{0}\right)}^{2}}{4q}\right\}$,$\;q=\frac{1}{w^{2}}+\frac{ik_{0}}{2R_{f}},$ $\psi_{p}\left(r\right)=\frac{J_{0}\left(\mu_{p}\frac{r}{R_{0}}\right)}{\left(\sqrt{\pi }R_{0}J_{1}\left(\mu_{p}\right)\right)}$.

**Figure 5:**
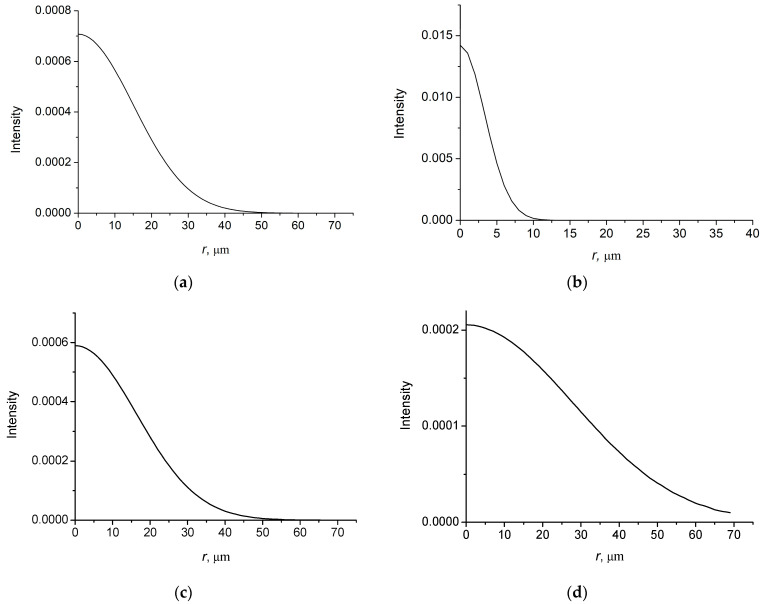
Intensity distributions of Gaussian beam with *w* = 30 μm and *λ* = 0.63 μm at different distances: (**a**) *z* = 0; *R*_f_ = 1000 µm; (**b**) *z* = 1000 µm, *R*_f_ = 1000 µm; (**c**) *z* = 2000 µm, *R*_f_ = 1000 µm; (**d**) *z* = 2000 µm, *R*_f_ = 100,000 µm.

Figure 5 shows the radial intensity distributions $I\left(r,z\right)={\left|E\left(r,z\right)\right|}^{2}$ of a Gaussian beam with the wavefront curvature radii *R_f_* = 1000 µm and *R_f_* = 100,000 µm at different propagation distances.

It can be seen that the beam width decreases significantly at a geometrical focusing plane *z* = *R*_f_ = 1000 µm. Note that tight focusing takes place if the wavefront curvature radius is not much different from the width of the incident beam. In this case, the nonparaxial effects become significant.

It is of interest to analyze the behavior of the beam profile near the focusing plane taking into account nonparaxial effects. It is known that in the paraxial approximation, the propagation of a Gaussian beam with the preservation of the profile is observed. However, shape-invariant propagation is disrupted due to nonparaxial effects.

**Figure 6:**
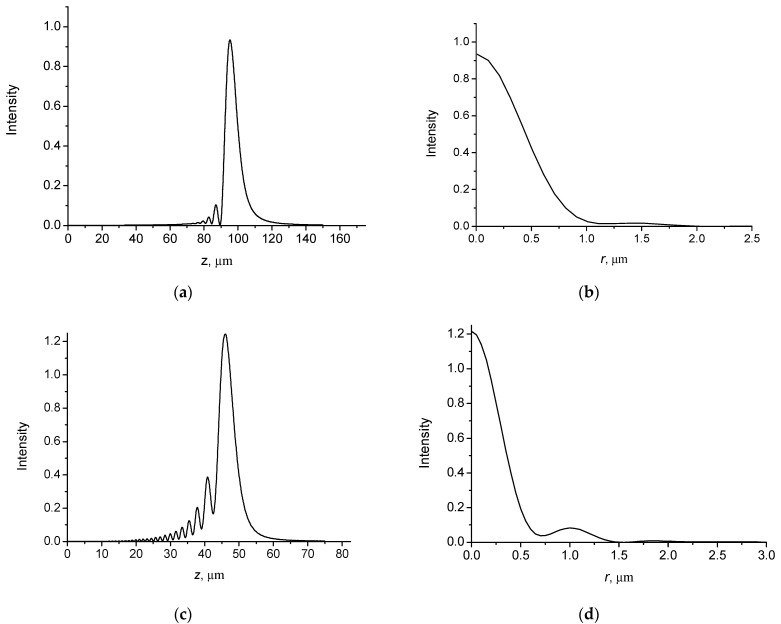
Intensity distributions of focused Gaussian beams in axial direction and radial direction at focus planes: (**b**) *z*_f_ = 95.3 µm; (**d**) *z*_f_ = 46.0 µm. Incident beams with *w* = 30 µm, *λ* = 0.63 µm and *R*_f_ = 100 µm (**a**,**b**) and *R*_f_ = 50 µm (**c**,**d**).

Figure 6 shows the changes in the intensity of the field with distance along the axial direction (Figure 6a,c) and the intensity distributions of the focused Gaussian beam in the focusing plane in the radial direction (Figure 6b,d).

It follows that the focal planes of the nonparaxial and paraxial beams do not coincide. The focusing plane is shifted in the opposite axial direction compared to the geometric focusing plane if nonparaxiality is taken into account. For an incident Gaussian beam with a width *w* = 30 µm and a radius of the wavefront curvature *R*_f_ = 100 µm, we obtain a displacement of the focal plane by 4.7 µm (Figure 6a). For an incident Gaussian beam with a radius of the wavefront curvature *R*_f_ = 50 µm, a displacement of the focal plane is 4.0 µm (Figure 6c). There is a significant difference in the axial intensity distributions in front of and behind the focus. Before the focus plane, there are significant oscillations in the field intensity. This asymmetry is caused by nonparaxiality. The beam intensity profile in the focus plane does not correspond to the Gaussian profile. It can be seen, that a noticeable sidelobe appears in the profile of the beam (Figure 6d). There is no sidelobe in the paraxial approximation. Note that these effects were also observed with nonparaxial focusing of light beams in a graded-index medium [36,37]. The observed effects may be important in optical trapping and manipulation of nanoparticles.

## 5. Conclusions

Thus, the nonparaxial evolution of a closed-form CSD of a Bessel-correlated beam in free space is represented by an incoherent superposition of diffraction-free Bessel modes. Instead of partially coherent beams with Laguerre–Gauss modes [24], here, we have considered a family of diffraction-free Bessel vortex beams. It is shown that the decomposition of arbitrary incident fields into Bessel beams with a truncated aperture is an effective method for analyzing nonparaxial propagation and tight focusing of light in free space.

It was shown in [24] that the cross-spectral density is shape invariant during propagation in free space, due to the fact that the LG modes are shape invariant on paraxial propagation. Our results show that shape-invariant propagation is not observed when nonparaxial effects become significant.

The observed nonparaxial effects (asymmetry in the intensity distribution in the axial direction and the appearance of sidelobes in the transverse field intensity distribution) at a beam focusing in free space were also shown earlier by numerical modeling [64]. Our results show that the beam profile of a partially coherent vortex beam can be shaped by changing its initial radius of wavefront curvature, which is useful for optical trapping.

Here, we have considered the nonparaxial propagation of scalar fields in free space. However, electromagnetic fields have a vector nature, and polarization effects play a significant role in the propagation of partially coherent fields. Therefore, it is of interest to extend the results to vector fields. This is especially important for describing tightly focused beams. The extension of coherence from the scalar to the vector domain can be formulated using generalized two-point Stokes parameters [65]. It is of fundamental and practical interest for studying the coherence-induced polarization effects in free space [66,67,68,69,70,71,72,73,74,75].

Future research may be related to the consideration of the propagation of vector vortex partially coherent and partially polarized beams. Of particular interest is the consideration of structured vortex flows with an orbital angular momentum [76,77,78,79,80,81,82] and the effects of nonparaxiality and depolarization during propagation [83,84]. Other important topics are the coherence and polarization effects in gradient-index medium [85], plasmonic structures [86,87], in turbulent media, etc.

In summary, it is shown that the coherent mode decomposition is an effective method for analyzing the nonparaxial propagation of partially coherent vortex beams in free space. A modified cross-spectral density function corresponding to the family of Bessel-correlated beams is introduced. Explicit analytical expressions for the mode decomposition weights of the CSD function are obtained. The possibility of analyzing nonparaxial propagation and focusing of Bessel–Gauss vortex beams in free space using the mode decomposition method is demonstrated. A noticeable asymmetry in the axial intensity distribution in front of and behind the focus caused by nonparaxiality is shown.

The results may be useful for trapping microparticles where a focused beam spot with a special beam profile is required, and may also be of interest for information transmission, optical imaging and free space optical communication.

## Data Availability

Not applicable.
